# Axonal Regeneration after Spinal Cord Injury: Molecular Mechanisms, Regulatory Pathways, and Novel Strategies

**DOI:** 10.3390/biology13090703

**Published:** 2024-09-07

**Authors:** Mohammed Ibrahim Elmalky, Gonzalo Alvarez-Bolado, Alexander Younsi, Thomas Skutella

**Affiliations:** 1 Institute for Anatomy and Cell Biology, Department of Neuroanatomy, Group for Regeneration and Reprogramming, Medical Faculty, University of Heidelberg, Im Neuenheimer Feld 307, 69120 Heidelberg, Germany; mohamed.ibrahim@uni-heidelberg.de (M.I.E.); alvarez@uni-heidelberg.de (G.A.-B.); thomas.skutella@uni-heidelberg.de (T.S.); 2 Department of Neurosurgery, Heidelberg University Hospital, Im Neuenheimer Feld 400, 69120 Heidelberg, Germany

**Keywords:** myelin-associated inhibitors (MAIs), neurotrophins, PKA/AMP, PI3K/Akt/mTOR, guidance cues, molecular and cellular mechanisms, axonal regrowth

## Abstract

**Simple Summary:**

The main challenge of axonal regeneration after traumatic injuries of the spinal cord consists of overcoming the activation of inhibitory pathways. Understanding the molecular mechanisms affecting regrowth (like the PKA/AMP, PI3K/Akt/mTOR pathways) and guidance cues (like neurotrophins) is thus essential. The use of gene therapy, tissue engineering, and small therapeutic molecules show a promising track to overcome inhibitory mechanisms to axonal regrowth.

**Abstract:**

Axonal regeneration in the spinal cord after traumatic injuries presents a challenge for researchers, primarily due to the nature of adult neurons and the inhibitory environment that obstructs neuronal regrowth. Here, we review current knowledge of the intricate network of molecular and cellular mechanisms that hinder axonal regeneration, with a focus on myelin-associated inhibitors (MAIs) and other inhibitory guidance molecules, as well as the pivotal pathways implicated in both inhibiting and facilitating axonal regrowth, such as PKA/AMP, PI3K/Akt/mTOR, and Trk, alongside the regulatory roles of neurotrophins and axonal guidance cues. We also examine current insights into gene therapy, tissue engineering, and pharmacological interventions that show promise in overcoming barriers to axonal regrowth.

## 1. Introduction

Traumatic spinal cord injuries (SCIs) occur in approximately 16 cases per million inhabitants in Europe, and over 27 million people globally suffer from such injuries [1,2]. It used to be that people aged 15–29 were primarily affected by SCI due to traffic accidents. In developing countries with a reported yearly occurrence rate of SCI of 25.5 per million, males with an average age of 32.4 years involved in vehicle collisions are still the predominant SCI victims [3]. More recently, however, a new peak has been observed in incidence among individuals over 70, mainly due to falls [1,4]. SCI leads to significant functional impairments, including motor, sensory, and autonomic deficits, potentially resulting in complete or partial paralysis. Secondary complications such as muscle spasticity, pressure sores, neuropathic pain, and psychological disorders arise frequently, enormously increasing the lifetime healthcare costs of the patients [5,6]. 

The main pathophysiological mechanisms behind such sequelae of SCI are well understood. The primary injury typically arises from an initial mechanical force exerted on the spinal cord tissue, leading to immediate disruption and displacement of the vertebral column and, subsequently, widespread cellular loss, axonal damage, and disruption of the vasculature and blood–spinal cord barrier. As a consequence, a cascade of deleterious events, collectively termed “secondary injury,” ensues within minutes of the initial insult, exacerbating spinal cord damage. Secondary injury encompasses various pathological processes such as vascular compromise, edema formation, ionic imbalance, excitotoxicity mediated by neurotransmitters, lipid peroxidation, generation of free radicals, influx of calcium ions, cellular demise, demyelination, and axonal degeneration, among others. These molecular and cellular alterations culminate in extensive neurodegeneration and disruption of spinal cord circuitry and connectivity, ultimately resulting in permanent neurological deficits following SCI. Spontaneous neurological recovery post-SCI is severely limited due to the progressive nature of tissue damage, extensive cell loss, and the inherent limited capacity of the adult spinal cord tissue for self-repair [7]. 

Nevertheless, as of the present time, a consensus on an acknowledged pharmacological intervention for spinal cord injury (SCI) has yet to be established. This underscores the formidable challenges inherent in developing therapeutic strategies capable of comprehensively addressing the diverse array of pathophysiological processes and cellular mechanisms involved in SCI. Consequently, the quest for a definitive pharmacological intervention remains an ongoing and evolving endeavor in the field of SCI research. For this reason, rehabilitation plays a crucial role in managing residual neurological functions and improving quality of life. However, the effectiveness of rehabilitation is challenging to prove scientifically, with few interventions supported by evidence [8,9]. Thus, a more profound understanding of the pathophysiology of SCI and the reasons for impeded neuronal regeneration, combined with emerging experimental treatments to improve neurological function, is still very much needed.

The historical understanding of SCI dates back over 5000 years, with treatments discussed in ancient Ayurveda and Egyptian medicine. Cervical SCI was addressed in ancient Egypt. Hippocrates and Galen contributed to SCI treatments in Greece and Rome. Centuries later, in 1890, German pathologist Hans Schmaus initiated the first animal model experiment for SCI in rabbits, and Goltz and Sherrington’s experiments on spinal shock in animals led to further insights into SCI-induced functional paralysis. Since then, various animal models, including rats, mice, dogs, zebrafish, and even salamanders (capable of limb regeneration after amputation), have been employed to elucidate the factors inhibiting axonal outgrowth in neurons [10]. 

Today, it is well established that the migration of microglia, astrocytes, and oligodendrocytes to the injury site forms a complex physical barrier preventing neurite regeneration [11]. An additional “chemical barrier” further contributes to the obstruction of axonal outgrowth. This barrier is formed by molecules like the Rho family of GTPases, PTEN, various cytokines (e.g., IL6, IL4), and myelin-associated inhibitors (MAIs) such as neurite outgrowth inhibitor A (Nogo-A), myelin-associated glycoprotein (MAG), and oligodendrocyte myelin glycoprotein (OMgp). A decrease in cAMP signaling resulting from the injury adds to the problem. For these reasons, addressing how the barriers originate and how to eliminate them has become a focal point of scientific inquiry.

While current research shows significant progress in restoring motor and functional abilities in animal models, achieving a complete and healthy recovery in human patients remains challenging. In this review, we concentrate on the signaling pathways and inhibitory factors key to unlocking axonal regeneration after SCI. We will also discuss recent advancements in gene therapy, tissue engineering, and pharmacological treatments for axonal regeneration.

## 2. Adult Central Nervous System (CNS) Response to Traumatic Lesions and Molecular Mechanisms of Axonal Regrowth Inhibition

In contrast to the peripheral nervous system, the adult CNS does not exhibit natural regeneration after injury. 

Axon outgrowth involves the extension of actin filopodia and lamellipodia. During development, axonal elongation is facilitated by attraction cues, like Netrin-1, neurotrophins, and Laminin, which guide neurites to their intended targets [12]. Upon axonal lesion, the reverse process ensues. The dynamic depolymerization of microtubules and actin filaments leads to the collapse of filopodia and lamellipodia and subsequent loss of tension and adhesion to the surrounding cells; Myosin II, activated by Rho kinases, blocks the plus ends of microtubules, causing axonal shrinkage and retraction via a retrograde f-actin flow; finally, repulsion cues, including ephrin A2, ephrin A5, and semaphorin A3, further contribute to inhibiting growth cones and axons [13]. RhoA-induced repulsion inhibits actin formation, increases growth cone contractility, diminishes axonal and growth cone adhesion, and activates ephrin A2 and semaphorin A3, promoting endocytosis and inhibiting integrin receptors, ultimately leading to axonal retrusion [13]. PTEN activation after injury also triggers GSK3 kinase, which is necessary for semaphorin A3 activation, resulting in f-actin unit collapse and growth cone retreat [14]. The myelin-associated inhibitors like Nogo, MAG, and OMgp are relevant to inhibiting axonal regeneration following injury [11,15].

Kinases also play a vital role in neurite formation and axonal regrowth by activating or inactivating their pathways, leading to cytoskeletal rearrangement, increased neural surface area, differentiation, and metabolic regulation. Essential kinases involved include protein kinase B (Akt), glycogen synthase kinase 3 beta (GSK3β), mammalian target of rapamycin (mTOR), and protein kinase A (PKA) [12].

Shifting the focus to the physical barriers, glial scar formation commences shortly after injury, with substantial astrocyte proliferation occurring within the first week and gradually decreasing over subsequent weeks [16,17]. Other components of the glial scar, including macrophages and microglia, also hinder axonal regeneration [17]. 

In summary, the natural regeneration of adult CNS axons faces hurdles due to biochemical inhibitors and physical barriers. While, during development, attractive cues like Netrin-1, neurotrophins, and Laminin guide axonal elongation, this process reverses post-lesion. The depolymerization of microtubules and actin filaments causes the collapse of filopodia and lamellipodia, inducing axonal shrinkage and retraction mediated by activated Myosin II through Rho kinases. Repulsive cues like ephrin A2, ephrin A5, and semaphorin A3 further impede axonal growth. Myelin breakdown releases inhibitors, such as Nogo, MAG, and OMgp, hindering axonal regrowth. Kinases like Akt, GSK3β, mTOR, and PKA regulate neurite formation and axonal regrowth by modulating cytoskeletal rearrangement and metabolic regulation. Glial scar formation, beginning shortly after injury, involves robust astrocyte proliferation, peaking within the first week and gradually decreasing over subsequent weeks. Additionally, components like macrophages and microglia hinder neuronal axon regeneration.

## 3. Molecular and Biochemical Factors Obstructing Axonal Regeneration after SCI

### 3.1. Myelin-Related Factors Preventing Axonal Regeneration

Some of the main inhibitors of axonal and growth cone regeneration are myelin-derived molecules, such as neurite outgrowth inhibitor A (Nogo-A), myelin-associated glycoprotein (MAG), and oligodendrocyte myelin glycoprotein (OMgp) [18]. 

#### 3.1.1. Neurite Outgrowth Inhibitor A (Nogo-A)

Nogo-A belongs to a membrane protein family including Nogo-B and -C. It is about 200 kDa and 195 amino acids long and is expressed in oligodendrocytes [19]. Treatment with an anti-Nogo antibody has shown promise in regenerating damaged areas and improving motor function recovery [20]. Blocking Nogo-A after SCI in adult rats requires approximately 10 to 12 days for treatment efficacy, with similar expectations for human patients [21].

#### 3.1.2. Myelin-Associated Glycoprotein (MAG)

MAG, a member of the immunoglobulin Ig-like family [22], is expressed in Schwann cells, oligodendrocytes, and segments of the myelin sheath [23]. It has an essential role in the myelin inhibition of developing neurons [24].

Recent studies have shown the relationship between neurons and MAG. In 2011, the group of Moon co-cultured rat cerebellar neurons on an inhibitory cellular substrate expressing MAG and found that MAG blocked neurite outgrowth [25]. This finding abolished the idea that MAG promotes axonal regeneration. The authors also found several deficiencies in the cAMP levels and the downregulation of PKA activity related to the change in MAG, as it is an inhibitor of axonal regeneration. The neuronal response to MAG and the role of MAG in growth inhibition in the CNS were also clarified. Further studies utilized MAG knockout mice to compare damaged and healthy white matter, confirming that wild-type MAG inhibits neurite regeneration [26]. These findings underscore MAG’s critical role in axon growth inhibition [27].

#### 3.1.3. Oligodendrocyte Myelin Glycoprotein (OMgp)

OMgp is a glycosylphosphatidylinositol (GPI) CNS myelin protein and a neurite outgrowth inhibitor. It is expressed in oligodendrocytes, neurons, astrocytes, and myelinated CNS axons [24].

OMgp expression levels increase during the developmental stages of myelination. OMgp acts as an inhibitor of axonal regeneration both in vitro and in vivo [28]. OMgp binds to Rho GTPases through NgR signaling to inhibit neuronal regeneration [29].

NgR is the receptor protein for the three isoforms of myelin-associated inhibitors, including Nogo-A, myelin-associated glycoprotein (MAG), and oligodendrocyte myelin glycoprotein (OMgp). The expression of NgR is one cause of the collapse of axonal regeneration in adult SCI. The downregulation and inhibition of RhoA increase axonal growth and regeneration after injury [30].

### 3.2. RhoA/ROCK and Rac1

The RhoA/ROCK protein family consists of the three GTPases: Cdc42, Rac, and Rho. These enzymes regulate the actin–myosin cytoskeleton and are essential for processes involving changes in cell morphology, including neurite outgrowth and axonal collapse [31].

Rho exists in two forms: the inactive form bound to GDP (Rho-GDP) and the active form bound to GTP (Rho-GTP) [32]. There are several Rho proteins with different names like Rho A, B, and C. RhoA is closely involved in various cellular processes, such as transcription, cell transport systems, axonal microtubule motion, cytokine formation, cell movement, cell–cell contact complexes, and neural outgrowth. It also plays a role in stress fibers that regulate filo- and lamellipodia, the movement-responsible parts of neurites. Rho guides post-synaptic regions during axon regeneration [33]. As a consequence, RhoA is involved in changes in growth cone structure and behavior, including those leading to collapse and axonal retrusion [33]. The downregulation of the Rho-activating enzyme Rho-kinase has been shown to suppress repulsive mechanisms in the CNS of rats and frogs [33]. In the same direction, the downregulation of the RhoA function by dominant negative RhoA supports growing growth cones with robust filopodia [31]. 

Rac1 regulates actin network branching and positively impacts axonal outgrowth, triggering attractive guidance cues and contributing to growth cone formation. Rac1 upregulation enhances axonal regeneration activity [33]. The expression of constitutive active (CA) RhoA and CA Rac1 regulate the up- or downregulation of RhoA and Rac1 [34]. CA Rac promotes neurite dynamics and motility, while dominant negative Rac has the opposite effect on neurite branching [35]. Rac1 reduces RhoA activity through mechanisms such as PAK kinase downregulation. Conversely, RhoA-GTP kinase downregulates Rac1, suppressing its activity by promoting effector kinases ROCK1/2 [36,37]. 

### 3.3. cAMP and PKA

cAMP signaling is essential in neural regeneration, contributing significantly to neurite outgrowth, axonal guidance, and the modulation of glial cell responses following SCI. Its influence extends to restraining and hindering immune cells, including macrophages/microglia, astrocytes, and oligodendrocytes after SCI, affecting the overall repair process. cAMP can also enhance the neuronal differentiation of neural stem or progenitor cells and their proliferation rate. cAMP upregulation in the growth cone impacts the upregulation of PKA, leading to a decrease in the Rho-ROCK pathway and promoting axonal outgrowth [38].

cAMP expression is upregulated during axonal elongation of neonatal dorsal root ganglion (DRG) neurons, both in vivo and in vitro, underscoring its critical role in growth cone dynamics and axonal regeneration [39]. 

Elevated cAMP levels in neurites correlate with increased production of neurotrophins, such as BDNF and NGF. These neurotrophins bind to the TrkB receptor, activating the PI3K/AKT/mTOR pathway and promoting axonal outgrowth. Moreover, heightened cAMP levels offer protection against myelin’s repulsive activity, which has a multifaceted impact on axonal regeneration [40,41].

However, MAG assumes a contrasting role by downregulating cAMP levels, leading to growth cone collapse and axonal repulsion [42].

Forskolin, a natural plant compound, increases PKA/cAMP pathway expression, inducing the differentiation and proliferation of neural stem cells. In various studies, forskolin increases neuronal survival and helps regeneration. A study by Watanabe and colleagues in 2003 showed that the addition of forskolin increases cAMP levels to rapidly facilitate the translocation of TrkB receptors from intracellular stores to the plasma membrane. This enhances the survival of the retinal ganglion cells, and, in combination with different neurotrophins, increases the neuronal survival rate [43].

In summary, cAMP signaling emerges as a central regulator in the intricate interplay of molecular processes governing neurite outgrowth and axonal regeneration, with diverse and critical roles in neural repair. 

### 3.4. PI3K/Akt/mTOR and PI3K/Akt/GSK3β Signaling Pathways

The significance of the PI3K/Akt/mTOR pathway extends beyond its involvement in neuronal degeneration; it also plays a crucial role in regeneration, enhancing neuronal and functional recovery following injury [44]. Both the PI3K/Akt/mTOR and PI3K/Akt/GSK3β signaling pathways exert a direct impact on the RhoA/ROCK pathway, influencing growth cone collapse and axonal regeneration within the CNS [45].

Neurotrophins like BDNF and elevated PKA levels contribute to the activation of the PI3K/AKT pathway, subsequently downregulating GSK3β [46]. This pathway is crucial for neuronal survival and neurite outgrowth, with PTEN negatively impacting neural outgrowth by suppressing the PI3K/AKT pathway and activating GSK3β, leading to mTOR inhibition [47]. The PI3K/AKT pathway is activated by neurotrophins, like NGF and BDNF, through the TrkB receptor [48]. The mediation of the neurotrophins also leads to the activation of the cAMP/PKA and Rac/WAVE pathways, which are known to promote neuronal outgrowth and survival [49].

The PI3K/AKT pathway is strongly associated with cell and neurite survival, with its modulation by GSK3β inhibition. Conversely, the activation of GSK3β leads to upregulated Caspase-3 expression, resulting in cell damage and apoptosis [50]. 

Numerous studies consistently demonstrate that the activation of the PI3K/AKT pathway inhibits GSK3β, contributing to neural survival and regeneration in vivo experiments spanning various neurodegenerative diseases and CNS traumas [51]. 

Additionally, increased cAMP levels in neurons activate the PKA pathway and trigger the PI3K/AKT pathway, upregulating mTOR expression and facilitating neural cell survival and regeneration [51].

**Figure 1:**
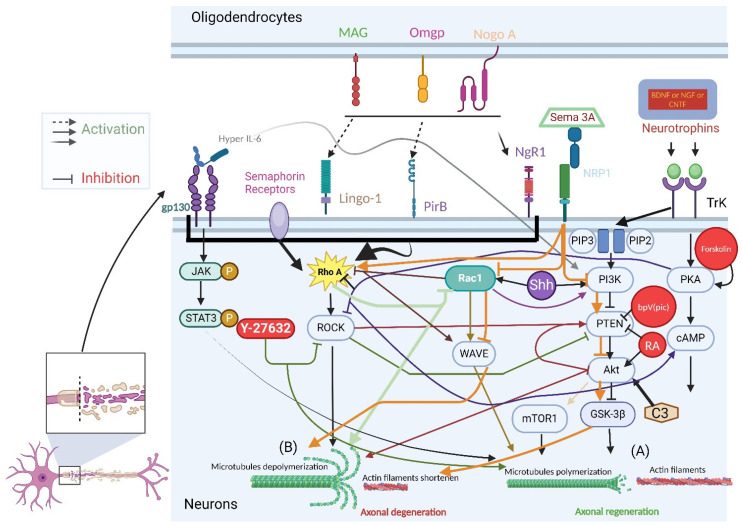
Molecular mechanisms of inhibitory and repulsive molecules in SCI leading to axonal cytoskeletal collapse and therapeutic strategies utilizing activatory and attractive molecules for regeneration pathway activation and cytoskeletal reconstruction (created with BioRender.com). (**A**) Axonal growth involves microtubule polymerization in axons and the formation of growing actin filaments, which leads to the formation of growth cones. (**B**) Axonal growth inhibition includes microtubule depolymerization in axons and actin filament retrusion, resulting in growth cone collapse. Myelin receptors on the oligodendrocytes, like myelin-associated inhibitors neurite outgrowth inhibitor A (Nogo-A), oligodendrocyte myelin glycoprotein (Omgp), and myelin-associated glycoprotein (MAG), and all three ligands signal through the different neuronal receptors like Nogo receptor 1 (NgR1), Leucine-rich repeat and Ig domain containing 1 (Lingo-1), and paired immunoglobulin-like receptor B(Pir-B) activating the Rho A/ROCK pathway during SCI. This reduces actin filaments and causes microtubule depolymerization. Y-27632 inhibits the Rho A/ROCK pathway in injured neurons, facilitating neurite regeneration (**A**). Neurotrophins such as BNDF, NGF, and CNTF bind to TrkB receptors, activating PKA and increasing cAMP levels, promoting the (**A**) pathway. Forskolin elevates PKA levels, diminishing Rho A/ROCK pathway activity and boosting cAMP levels, further supporting regeneration (purple) and facilitating neurite regeneration (**A**). Rac1 activation, either by Rho A/ROCK inhibition (brown) or Sonic hedgehog (Shh) stimulation, fosters the Rac1/WAVE pathway, facilitating regeneration (light brown) and neurite regeneration (**A**). The expression of Rac1 has a good impact on lowering the axonal outgrowth inhibitors. By expressing RhoA/ROCK, the Rac1 pathway is suppressed (light green). The interaction between PI3K/Akt/mTOR and PI3K/Akt/GSK3β signaling pathways directs the catalytic conversion of phosphoinositol phosphatase 3 (PIP3) to phosphoinositol phase 2 (PIP2) by PI3K and phosphatase and tensin homolog (PTEN) inhibition that activates AKT, which, in turn, induces the mammalian target of rapamycin (mTOR) (gold). Alternatively, PI3K and phosphatase and tensin homolog (PTEN) inhibition activates AKT, which, in turn, inhibits glycogen synthetase kinase 3 beta (GSK3b), which expresses cyclic adenosine monophosphate(cAMP) and turns on PKA, which inhibits Rho and Rho-kinase facilitating neurite regeneration (**A**). Retinoic acid (RA) is utilized to inhibit PTEN, activate AKT, inhibit GSK3b, activate mTOR, and promote C3, thereby facilitating regeneration. The Rho A/ROCK inhibitor Y-27632 also acts on injured neurons by inhibiting the Rho A/ROCK pathway (green), suppressing PTEN activation and AKT activation, ultimately preventing GSK3b activation or promoting mTOR. Activation of the Rho A/ROCK pathway leads to PTEN activation and AKT inhibition, which, in turn, inhibits both the PI3K/Akt/mTOR and PI3K/Akt/GSK3β signaling pathways, resulting in (**B**) pathway and axonal degeneration (dark red). Conversely, the binding of Hyper-IL-6 with the gp130 receptor triggers the activation of JAK/STAT3 and PI3K/AKT/mTOR signaling via PTEN downregulation (gray), leading to the (**A**) pathway. Semaphorin 3A (Sema 3A) repulses axons through the co-receptor protein neuropilin-1 (NRP1) of the inhibiting PI3K/Akt/GSK3β and Rac1/WAVE signaling pathways. This results in the inhibition of axonal growth and activation of the Rho A/ROCK pathway (orange), ultimately leading to axonal growth inhibition pathways (**B**) and axonal degeneration.

In summary, the PI3K/Akt/mTOR pathway is vital for neuronal regeneration, influencing growth cone collapse and axonal regeneration in the CNS. The inhibition of GSK3β via this pathway enhances cell survival, while its activation leads to cell damage. PI3K/AKT activation (for instance by increased cAMP) inhibits GSK3β, aiding neural regeneration in various conditions (see Figure 1 below).

### 3.5. Phosphatase and Tensin Homolog (PTEN)

PTEN, a critical regulator in neural regeneration, has been the subject of various in vitro and in vivo studies. Findings consistently show that inhibiting PTEN promotes axonal regeneration and elongation. Liu and colleagues demonstrated this phenomenon, emphasizing the significance of PTEN/Akt/mTOR signaling pathway activation, particularly in cerebral cortical neurons [52]. PTEN’s negative influence manifests in removing D3 from phosphatidylinositol trisphosphate (PIP3), hindering the activation of the PI3K/Akt/mTOR pathway [52]. In a study by Leibinger and colleagues in 2021, the knockout of PTEN in cortical neurons resulted in the activation of the PI3K/AKT/mTOR pathway, ultimately promoting axonal regeneration [53].

Various PTEN inhibitors have been developed, including bpV(pic), SF1670, and VO-Ohpic trihydrate. The PTEN-inhibitory efficacy of bpV(pic) has been evaluated, demonstrating its ability to suppress PTEN expression effectively [52]. Upon the addition of bpV(pic), the phosphorylation levels of Akt and p70S6K (Thr421/Ser424) significantly increased compared to the control group. Furthermore, there was a slight elevation in the phosphorylation levels of mTOR. Although no apparent dose-dependent effects were observed for bpV(pic), the inhibitory efficiency was still evident when neurons were cultured until day 14. This underscores the sustained effectiveness of bpV(pic) in inhibiting PTEN, which has been shown to promote axonal regeneration through the PI3K/Akt/mTOR pathway [52]. 

In summary, studies confirm PTEN’s negative impact on neural regeneration in the CNS. The PTEN inhibitor bpV(pic) promotes axonal regeneration by activating the PI3K/Akt/mTOR pathway.

### 3.6. JAK/STAT3 Pathway

Fischer and his colleagues in 2021 caused experimental SCI on PTEN knockout (PTEN−/−) mice. These mice recovered motility of both hind legs after being treated with hyper interleukin-6 (hIL-6). hIL-6 works by upregulating the JAK/STAT3 pathway, which is known to inhibit axonal regeneration inhibitors and promote neuron regeneration and survival [53].

### 3.7. Glial or Perilesional Scar

The glial scar rapidly forms around CNS lesions within two to three weeks post-injury in rodents. This process is initiated by the breakdown of the blood–spinal cord barrier (BSCB) and the infiltration of the CNS parenchyma by non-neural cells. Initially, the infiltrated parenchyma at the lesion site shows edema, myelin debris, and the presence of degenerating neurons and glia. Over time, the edema diminishes, and a fibrotic scar develops, occupying the entire lesion epicenter by eight days post-injury in rodents. This scar consists mainly of invading hematogenous cells such as lymphocytes, macrophages, and leukocytes penetrating the spinal cord parenchyma [54]. Additionally, fibroblasts from damaged meninges migrate into the lesion core, where they proliferate and secrete molecules that alter the extracellular matrix (ECM) [55]. Glial cells are scarce within this fibrotic tissue; astrocytes near the lesion undergo hypertrophy over a few days, peaking at 2–3 weeks post-injury, contributing to the formation of the perilesional scar and separating its epicenter from the spared nervous tissue [56].

### 3.8. Inflammatory Mediators beyond the Lesion in the Spinal Cord

**Figure 2:**
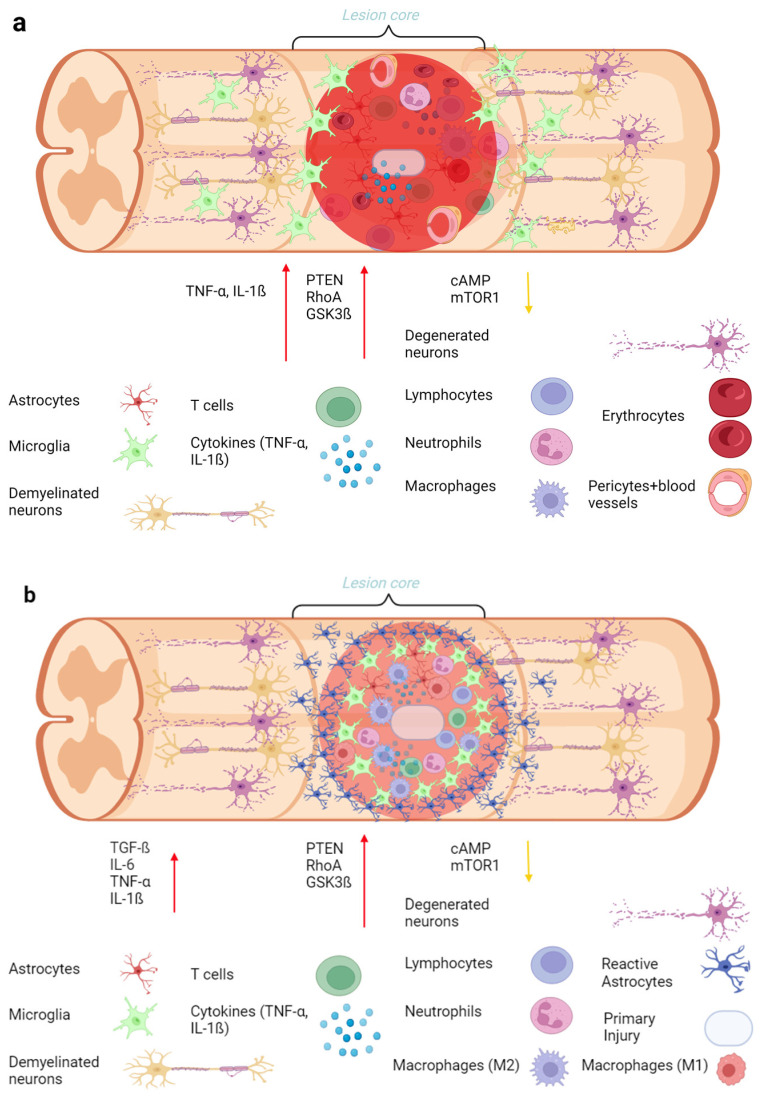

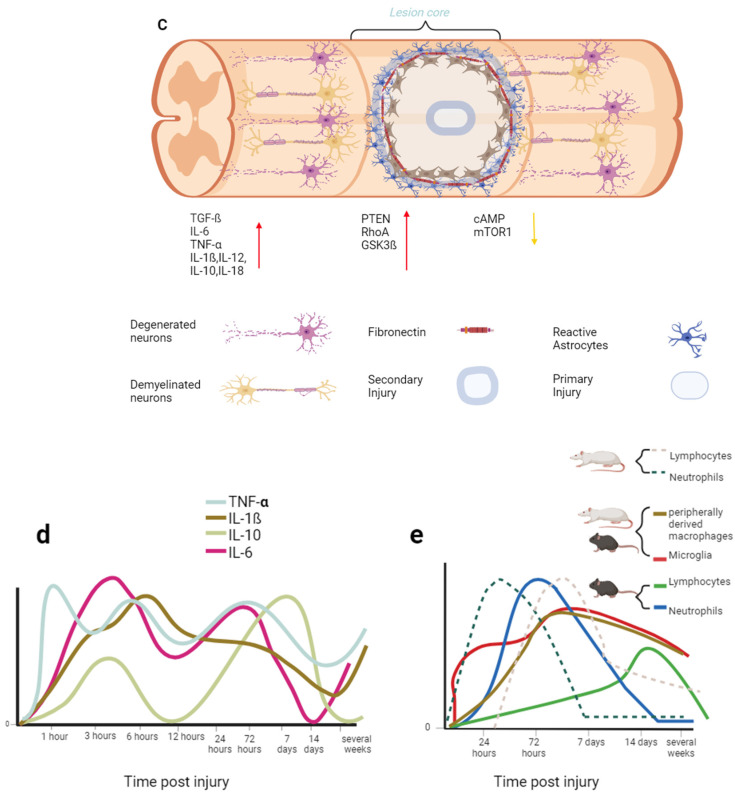
Schematics of the lesion site illustrating the various components of the glial scar following mechanical SCI in rodents (created with BioRender.com). (**a**) Schematic of the lesioned spinal cord acute phase (minutes to hours post-injury). The level of cellular responses shows neuronal damage with immediate necrosis of neurons and axons at the injury site. Disruption of the blood–spinal cord barrier leads to hemorrhage and edema and also highlights the rapid influx of neutrophils, T-cells, macrophages, and microglia. Cytokines and chemokines release pro-inflammatory cytokines (e.g., TNF-α, IL-1β), and chemokines attract immune cells to the injury site. Glutamate is released and causes excitotoxic neuronal death. Furthermore, several factors that inhibit neuronal regeneration, such as PTEN, GSK3ß, and Rho kinase pathways, are upregulated, while survival and proliferation factors, like cAMP and mTOR pathways, are downregulated. (**b**) In the schematic of the lesioned spinal cord of the subacute/intermediate phase (days to months post-injury), microglia and macrophages are activated and accumulated at the injury site and transition from a pro-inflammatory (M1) to an anti-inflammatory (M2) phenotype, participating in the phagocytosis of debris. Astrocytes begin to proliferate and form a glial scar, a process known as reactive astrogliosis. The continued expression of cytokines (e.g., IL-6, IL-1β) and growth factors (e.g., TGF-β) with the degradation of extracellular matrix (ECM) components and the reactive astrocytes contributes to the inhibition of the environment for axonal regeneration. Also, the presence of molecules like Nogo-A, MAG, and OMgp leads to axonal growth inhibition. Furthermore, several factors that inhibit neuronal regeneration, such as PTEN, GSK3ß, and Rho kinase pathways, are upregulated, while survival and proliferation factors, like cAMP and mTOR pathways, are downregulated. (**c**) Schematic of the lesioned spinal cord chronic phase (months to years post-injury). Glial scar maturation and stabilization of the scar, which permanently separates damaged from healthy tissue with limited and often aberrant sprouting of axons, with persistent activation of microglia and macrophages. Continuous expression of axonal growth inhibitors and ECM molecules. Ongoing production of inflammatory cytokines and chemokines like IL-1ß, IL-6, IL-12, IL-2, IL-18, and TNF-α. Scar-associated molecules stabilize the ECM components, such as fibronectin, which constitute the scar. Furthermore, several factors that inhibit neuronal regeneration, such as PTEN, GSK3ß, and Rho kinase pathways, are upregulated, while survival and proliferation factors, like cAMP and mTOR pathways, are downregulated. (**d**) The line graph illustrates the time pattern of cytokines, specifically IL-1ß, IL-6, IL-10, and TNF-α, following SCI. (**e**) Another line graph indicates the immune response and the increase in immune cells, including lymphocytes, neutrophils, and microglia, over time after SCI in different animals [61], modified from Hellenbrand et al., 2021 [65].

After SCI, various inflammatory mediators extend beyond the lesion site (see Figure 2 below), contributing to secondary damage. These mediators include cytokines, chemokines, and other pro-inflammatory molecules that exacerbate neuronal damage and impede recovery. Following SCI in mice, Fu et al. showed that there is an upregulation of surface antigens and a production of innate pro-inflammatory cytokines, including TNF-α, interleukin-1 (IL-1), interleukin-2 (IL-2), interleukin-6 (IL-6), interleukin-12 (IL-12), interleukin-10 (IL-10), interleukin-18 (IL-18), and chemokines, promoting further inflammation and neuronal apoptosis [57]. 

Infiltrating immune cells, including macrophages and lymphocytes, release additional inflammatory mediators, perpetuating a cycle of inflammation and tissue damage [58]. Understanding the role and regulation of these cells is crucial for developing therapeutic strategies to mitigate secondary injury and promote healing in the spinal cord [59,60].

Neutrophils invade the spinal cord, reaching their peak numbers within 24 h after injury. Subsequently, their numbers gradually decrease. Despite this decline, some neutrophils can stay in the injured spinal cord for as long as six months after SCI. Lymphocytes, on the other hand, typically begin to increase between three to seven days after SCI [60]. T cells also start to appear by this time and remain present until day 7. Macrophages and microglia show a strong presence by day 2 post-injury, with their numbers increasing between days 4 and 8. Astrocytes begin to populate the lesion core by day 7, and by the second week, the above-mentioned perilesional scar is formed through the actions of microglia and astrocytes (see Figure 2 below). From one month to four months post-injury, astrocytes produce proteins, such as collagen type IV and laminin, to seal the lesion core [61].

Regarding human SCI, only a few studies have explored this topic, such as Yang’s 2004 research which focused on the immediate immune, showing a significant increase in interleukin-1β (IL-1β), TNF-α, and IL-6 within 30 min post-injury. This was followed by the activation of microglia/macrophages approximately five hours after the injury. By the first day post-injury, neutrophils became widely distributed, and by the fifth day, macrophages, microglia, and neutrophils were all present within the lesion core [62]. Astrocytes, on the other hand, have a complex role because they accumulate around the injury site and proliferate to reduce the damage in the acute phase, while they obstruct endogenous cell migration during the subacute to chronic phases of SCI [63].

Oligodendrocytes are especially vulnerable to apoptotic loss, not only at the site of impact but also in areas distant from the epicenter of the SCI. This apoptotic loss results in the demyelination of surviving axons, both at the lesion epicenter and in surrounding regions. Fibroblasts invade the perilesional area and transform the extracellular matrix into fibrous connective tissue. This transformation includes the deposition of inhibitory extracellular matrix molecules, which serve as chemical barriers to axonal regeneration, akin to the inhibitors found in myelin [64].

## 4. Emerging Treatment Strategies to Overcome the Inhibition of Axonal Regeneration after SCI

### 4.1. Sonic Hedgehog and Retinoic Acid

Sonic hedgehog (Shh) is a secreted glycoprotein with signaling and morphogenetic functions. It has a vital role in the differentiation of the ventral CNS during development and plays an important essential role in neuron proliferation and survival. Shh acts through the Smo-Gli pathway to guide neurites by signaling and activating the src-family kinases to stimulate axonal outgrowth [66]. The expression of Shh after CNS injury helps the neural stem cells regenerate and survive [67]. 

Retinoic acid (RA) is an active form of vitamin A that is important during axonal regeneration. It has been shown that RA receptor β2 is involved in regenerating axons after SCI. Other experiments on DRGs using lentivirus RARβ2 expression showed an increase in the cAMP levels promoting increased axonal outgrowth. RA inhibits the PTEN after SCI, leading to an increase in the PIP3 via PI3K, which activates AKT, which, in turn, inhibits GSK-3, Bad, or Bax, which were activated during lesions. This process itself can activate the Raf, RacI, NF-κB, and mTOR pathways, which stimulate protein synthesis and ribosome biogenesis, as well as cell growth, which results in axonal regeneration [68].

### 4.2. KASH Proteins

The KASH membrane proteins (Klarsicht, ANC-1, Syne homology) are expressed on the outer membrane of the nuclear envelope and are connected to various cytoskeletal components, rendering them integral for actin machinery. Particularly significant in neurons, KASH proteins facilitate the transmission of compounds to cells through the cytoskeleton [69].

A groundbreaking study published in 2023 by Squair et al. analyzed the recovery of locomotion following paralysis. The research centered on the reinnervation of targets by the axons of injured spinal motoneurons treated with growth factors [70]. Utilizing a combination of GFP markers and KASH protein labeling of spinal cord nuclei, the authors identified the expression of homeodomain transcription factor Vsx2 (also called Chx10). Remarkably, Vsx2 was absent in healthy mice but present in those with SCI. These findings suggest that these neurons possess regenerative capabilities when exposed to targeted growth factors [70].

### 4.3. Stem Cell Transplantation

Another promising approach for CNS regeneration is the transplantation of exogenous stem cells, which are supposed to replace damaged tissue, including neurons, and promote axonal regrowth. Stem cell candidates are described in Table 1. 

When transplanted cells are introduced into the host’s CNS, many factors determine their successful integration. The transplanted cells must survive in the host environment, overcoming immune reactions, nutrient competition, and local environmental factors. Depending on the desired target tissue, the transplanted stem cells should differentiate into neurons to replace the damaged neurons or build intraneuronal connections between lesioned fibers. In addition, the transplanted neurons should show elongation of the axons and make synaptic connections to restore functional and locomotor recovery. This includes navigating the damaged part’s tissue and guiding it to reach its targets using attractive and repulsive cues. Finally, functional integration of the transplanted cells in the lesioned spinal cord network is essential. The cells should make connections and restore sensory and motoric functionality [71]. 

#### 4.3.1. Mesenchymal Stem Cells (MSCs)

The role of MSCs in SCI treatment shows considerable promise. MSCs exert their therapeutic effects through various mechanisms, including immunosuppression achieved via direct interaction with immune cells or the release of signaling molecules that mitigate inflammatory responses at the site of SCI [72]. Moreover, MSCs secrete neurotrophic factors, such as the brain-derived neurotrophic factor (BDNF) and the β-nerve growth factor (β-NGF), which facilitate axon regeneration. Additionally, MSCs modulate signaling pathways to inhibit glial scarring, a process that impedes axonal regrowth. Additionally, by mitigating glial scarring, MSCs create a conducive environment for axon regeneration [73].

Preclinical studies in SCI mice have demonstrated the beneficial effects of MSC treatment on motor function recovery. MSC therapy has been applied to patients with SCI, showing promising results. It has been observed to improve the sensory and motor scores recommended by the American Spinal Cord Injury Association, to facilitate bladder function recovery, and to promote neurological symptom alleviation [74]. Although some mild adverse effects like fever, gastrointestinal issues, headache, and urinary tract infections have been reported post-MSC treatment, they did not result in serious complications. Despite the encouraging outcomes observed in patients, the efficacy of MSC therapy reported in the literature is not yet sufficient to advocate for its widespread clinical application. Further improvements are needed to optimize the therapeutic potential of MSCs in SCI treatment. Additionally, numerous unanswered questions remain regarding the use of MSCs in SCI therapy, highlighting the necessity for continued research in this field [73].

#### 4.3.2. Induced Pluripotent Stem Cells (iPSCs)

Induced pluripotent stem cells (iPSCs) represent a breakthrough in stem cell technology, offering a solution to ethical concerns surrounding the use of embryonic stem cells. They are derived from adult somatic cells through artificial induction. The transplantation of iPSC-derived cells, embryonic stem cells (ESCs) [75], and iPSC-derived neural stem cells (NSCs) has emerged as prominent choices for SCI treatment.

In vivo models, iPSC-derived NSCs have demonstrated promising outcomes. They have been shown to decrease pro-inflammatory cytokine levels following SCI, thereby reducing the formation of glial and fibrotic scars in acute mouse models of thoracic SCI. Moreover, iPSC-derived NSCs exhibit the ability to promote the recovery of limb function. These findings underscore the therapeutic potential of iPSC-derived NSCs in the context of SCI and highlight their role in attenuating inflammatory responses and facilitating functional restoration [76].

### 4.4. Gene Therapy

Gene therapy offers a promising avenue to overcome impaired neural regeneration after SCI by targeting key molecules and pathways involved in axonal regrowth inhibition (Table 2). 

Such targets have been the prominent inhibitors of axonal growth Nogo, MAG, and OMgp, which contribute to growth cone retrusion and stalling. By targeting their downstream effectors, such as RhoA and ROCK, gene therapy can attenuate their inhibitory effects on axonal growth and foster regeneration. The RhoA/ROCK pathway role is pivotal in growth cone collapse and axonal growth inhibition. Gene therapy interventions targeting RhoA or ROCK inhibition can alleviate growth cone collapse and enhance axonal regeneration. By modulating the activity of RhoA and ROCK, gene therapy can promote cytoskeletal remodeling and facilitate axonal elongation, thereby overcoming regrowth inhibition [77].

The PTEN/mTOR signaling pathway regulates neuronal growth and promotes regeneration. Gene therapy approaches to downregulate PTEN expression can activate the mTOR pathway, leading to enhanced axonal regeneration. By manipulating the balance between PTEN and mTOR signaling, gene therapy can potentially augment CNS regeneration and recovery following injury or disease [78]. Cyclic adenosine monophosphate (cAMP) is a key intracellular signaling molecule in neuronal growth and plasticity. Gene therapy strategies to enhance cAMP levels offer a multifaceted approach to promoting axonal regrowth. By modulating cAMP-dependent pathways, gene therapy can potentiate beneficial signaling cascades while inhibiting adverse pathways, facilitating axonal regeneration and recovery. The neurotrophic factors, including ciliary neurotrophic factor (CNTF), brain-derived neurotrophic factor (BDNF), and nerve growth factor (NGF), play vital roles in promoting neuronal survival, growth, and regeneration. Gene therapy interventions aimed at upregulating the expression of these neurotrophic factors hold promise for enhancing axonal regrowth and facilitating recovery following neural injury or degeneration [78].

It is often argued that gene therapy represents a powerful tool for overcoming regrowth inhibition and promoting axonal regeneration in the CNS. Gene therapy interventions hope to restore neural connectivity and function in the face of injury or disease by targeting inhibitory molecules and modulating key signaling pathways. Continued research and development in this field hold promise for advancing the field of neural regeneration and improving outcomes for patients with neurological disorders.

From a technical point of view, a spectrum of gene therapy mechanisms stands at the forefront of scientific innovation. Harnessing the power of genetically engineered viruses, like adeno-associated viruses, lentiviruses, or retroviruses, the method of Viral Vector Transfection delivers tailored genes and factors to specific targets within the CNS [79]. These versatile viral vectors can be manipulated to carry genetic payloads encoding neurotrophic factors, inhibitory factor modulators, and signaling pathway modifiers, all geared towards fostering axonal regrowth. Operating on the principle of introducing small interfering RNA (siRNA) or short hairpin RNA (shRNA) molecules, the technique of RNA interference (RNAi) zeroes in on obstructive messenger RNA (mRNA) molecules. By selectively suppressing the expression of proteins, such as Nogo, MAG, or other inhibitory factors, RNA interference clears the path for CNS regeneration [80].

At the frontier of gene editing, the CRISPR/Cas9 system precisely alters target genes within cells, offering unprecedented control over genetic sequences [81]. By manipulating specific sequences that influence axonal regrowth, CRISPR/Cas9 can suppress inhibitory factors or amplify the activity of growth-promoting factors, paving the way for neural regeneration. Crafted from short, synthetic DNA or RNA molecules, Antisense Oligonucleotides (ASOs) are designed to bind to targeted mRNA molecules precisely. This binding impedes mRNA translation, thereby modulating the expression of inhibitory factors, such as Nogo or MAG, and suppressing or enhancing their effects on CNS regeneration. Leveraging the potential of modified stem cells, the Genetically Manipulated Cell Transplantation approach involves ex vivo manipulation of cells to augment neurotrophic factors and suppress inhibitory factors. These genetically enhanced cells are then transplanted into the injured site, seamlessly integrating into the tissue, promoting neuronal regeneration, and facilitating functional recovery [80].

Each of these gene therapeutic techniques has its advantages and challenges, and scientists are actively investigating them to use them to promote axonal outgrowth and overcome the inhibitory factors in the CNS after an injury.

### 4.5. Application of Small Molecules

#### 4.5.1. Small Molecules Promoting Axon Elongation

Small biochemical molecules are a promising way to promote axon regeneration in the CNS due to their ability to alter the cellular signaling pathways and gene expression and target extrinsic and intrinsic factors related to axonal outgrowth inhibition [82]. 

Epothilone is a microtubule-stabilizing agent that reduces growth cone collapse and enhances axon growth [83]. Epothilone promotes axon regeneration through its effects on microtubule stabilization and dynamic microtubule growth [84]. The application of epothilone for axon elongation has been investigated in various experimental setups, and the concentrations used may vary, depending on the specific model and the desired outcome in a study [83]. Epothilone B was applied at 1–10 nM in cell culture and 10 μg/kg in mice with SCI to promote axon growth and functional recovery.

RhoA and ROCK are the leading causes of growth cone collapse, axon outgrowth inhibition, and CNS neural degeneration. The RhoA/ROCK inhibitor Y-27632 (Stem cell Technologies, Cologne, Germany) has been shown to stop the growth cone collapse and promote axonal outgrowth in the presence of inhibitory factors, like MAIs [85].

Recombinant protein Cethrin inhibits the RhoA pathway in growth cone collapse and axon growth inhibition [86]. After SCI, its application has shown promise in promoting axon regeneration in animal models [87].

#### 4.5.2. Small Molecules Enhancing Intrinsic Neuronal Growth Potential

Forskolin, a potent adenylyl cyclase activator, elevates intracellular cyclic AMP (cAMP) levels, culminating in protein kinase A (PKA) activation. As shown by Miller 2002 [88], this cascade augments neuronal growth potential. Moreover, Filbin and his group in 2002 [89] elucidated the synergistic effects of forskolin in upregulating cAMP levels, thereby promoting axon regeneration and increasing neurotrophic factor expression within the CNS.

PTEN inhibitors are emerging as pivotal players in axonal regeneration. They modulate the phosphoinositide 3-kinase (PI3K) and mammalian target of rapamycin (mTOR) signaling pathways [90], facilitating axonal regeneration in CNS neurons. Additionally, in vivo models by He and colleagues in 2008 [91] demonstrated the efficacy of PTEN deletion or knockdown in promoting axonal outgrowth and neural regeneration following SCI.

The manipulation of epigenetic factors offers a promising avenue for CNS regeneration. Histone deacetylase (HDAC) inhibitors, such as trichostatin A (TSA) and valproic acid (VPA), modulate chromatin structure and gene expression, promoting axonal outgrowth by facilitating the expression of growth-enhancing genes [92]. Similarly, DNMT inhibitors, like 5-aza-2′-deoxycytidine (5-aza-dC) and Zebularine, exert their effects by demethylating DNA, thus enhancing gene transcription and axonal growth [93].

Modifying neurotrophic factor signaling pathways represents another crucial facet of neural regeneration. TrkB agonists, such as 7,8-dihydroxyflavone (7,8-DHF), enhance axonal outgrowth and neuronal survival by upregulating TrkB signaling, a receptor for brain-derived neurotrophic factor (BDNF) [94]. Additionally, GDNF-related compounds, including XIB4035 and GDNF family receptor α1 agonists, activate the GDNF pathway, promoting neuronal survival and axonal regeneration post-CNS injury [95].

#### 4.5.3. Synergistic and Combinatory Use of Small Molecules

Combining these small biochemical molecules to activate several signaling pathways can enhance axonal regeneration and improve functional recovery in CNS injury models; for example, combining cAMP and RhoA/ROCK inhibitors and neurotrophic factors can lead to massive axonal regeneration in the adult CNS [96]. In conclusion, utilizing tiny molecules to target intrinsic and extrinsic growth-inhibitory factors can offer significant advantages for promoting axon regeneration in the CNS. These molecules can potentially be developed into CNS-directed regenerative therapies, either as standalone treatments or in combination with other strategies, such as transcription factor manipulation or stem cell therapy. Further research is needed to identify novel small molecules, optimize dosages, and develop targeted delivery systems for maximal effectiveness and minimal side effects in treating CNS injuries and diseases.

## 5. Conclusions

In summary, the understanding of axonal and growth cone regeneration in the CNS has uncovered a multifaceted landscape of inhibitory and stimulatory factors. Research into these factors has not only elucidated their mechanisms of action but has revealed potential strategies for therapeutic intervention.

By combining direct reprogramming with other techniques and harnessing the potential of transcription factors, novel treatments for SCI could be developed. The inhibition of axonal regeneration after SCI involves complex mechanisms and pathways mediated by myelin-associated inhibitors (MAIs), like Nogo-A, MAG, and OMgp, along with other pathways such as Rho GTPases, cAMP signaling, PI3K/Akt/mTOR, and JAK/STAT3. Emerging treatment strategies offer promising approaches for promoting neural regeneration by enhancing cAMP signaling, inhibiting PTEN, and modulating the JAK/STAT3 pathway. Small molecules like Sonic Hedgehog, retinoic acid, and KASH proteins show potential in restoring motor function and promoting axonal regrowth. Small biochemical molecules like epothilone, Y-27632, cethrin, forskolin, PTEN, HDAC, DNMT, and neurotrophic factor modulators enhance axonal outgrowth and spinal cord repair. Combination therapies using these molecules may have a promising effect on axonal regeneration. Further research is necessary to optimize dosages, identify novel small molecules, and develop targeted delivery systems. Stem cells present additional opportunities for spinal cord neuron regeneration, provided they can effectively differentiate into neurons after being transplanted into the injury site. They extend axons and establish synaptic connections within the spinal cord network.

Furthermore, the integration of other various therapeutic strategies, including pharmacological interventions, gene therapies, and tissue engineering approaches, underscores the interdisciplinary nature of research in neural regeneration. Collaborative efforts across disciplines will be essential for translating scientific discoveries into clinically effective treatments that can improve patient outcomes and enhance the quality of life for individuals affected by SCI and diseases.

This quest holds immense promise for developing novel treatments for spinal cord injuries, neurodegenerative disorders, and other neurological conditions characterized by impaired axonal regeneration.

In essence, while the challenges of axonal regeneration in the CNS are formidable, the depth of understanding gained from research into inhibitory factors and signaling pathways offers hope for the development of innovative therapies that can unlock the regenerative potential of the nervous system and transform the landscape of neurological medicine.

## Figures and Tables

**Table 1:** Stem cell therapy for CNS regeneration involving different types of stem cells.

Different Stem Cell Types	Potency	Function
Embryonic stem cells (ESCs)	Multipotent stem cells, capable of transforming into many other cell types, are found in the embryo’s inner cell mass in its early developmental stage.	Using different factors, the ESCs can differentiate into any cell type, including neurons and glial cells.
Neural stem cells (NSCs)	Multipotent stem cells can be found in the embryonic and adult nervous systems.	NSCs can transform into different neural cell types, including neurons, astrocytes, and oligodendrocytes, making them suitable for CNS regeneration.
Mesenchymal stem cells (MSCs)	Multipotent stem cells can be found in bone marrow, adipose tissue, and other tissues.	They can differentiate into different cell types. They also have immunomodulatory functions, helping the CNS to recover by supporting its environment for tissue repair and regeneration.
Induced pluripotent stem cells (iPSCs)	Somatic adult cells undergo genetic reprogramming by Yamanaka’s four factors to resemble embryonic stem cells in their polarity. These cells are pluripotent and can differentiate into many cell types, including neural cells.	Derived from adult tissues, they present fewer ethical concerns than embryonic stem cells and can be patient specific, reducing the risk of immune rejection.

**Table 2:** Target molecules and signaling pathways are used in the gene therapy approach to overcome regrowth inhibition or activation.

Pathways	Mechanism of Action	Therapeutic Mechanism
Myelin-associated inhibitors (MAIs)	Nogo-A, MAG (myelin-associated glycoprotein), and OMgp (oligodendrocyte myelin glycoprotein) impede axonal growth.	Aimed at interrupting their associated signaling pathways, it can promote regrowth.
RhoA/ROCK (Rho-associated protein kinase)	The pathway is activated in response to MAIs and contributes to growth cone collapse and axonal growth inhibition.	Inhibiting RhoA or ROCK can improve axonal regeneration.
PTEN/mTOR (phosphatase and tensin homolog/mammalian target of rapamycin)	The signaling pathway plays a critical role in regulating neuronal growth and regeneration.	The downregulation of PTEN can enhance regenerative capacity by activating the mTOR pathway.
cAMP (cyclic adenosine monophosphate)	An important intracellular signaling molecule involved in neuronal growth and plasticity.	Enhancing cAMP levels can override growth inhibition imposed by inhibitory factors.
Neurotrophic factors (ciliary neurotrophic factor (CNTF), brain-derived neurotrophic factor (BDNF), and nerve growth factor (NGF))	It interacts with specific receptors on neurons and plays crucial roles in promoting neuronal survival, growth, and regeneration.	Upregulating their expression can enhance axonal regrowth and has therapeutic potential for nervous system recovery.

## Data Availability

No new data were created or analyzed in this study. Data sharing is not applicable to this article.
